# Effects of homework creativity on academic achievement and creativity disposition: Evidence from comparisons with homework time and completion based on two independent Chinese samples

**DOI:** 10.3389/fpsyg.2022.923882

**Published:** 2022-08-12

**Authors:** Huiyong Fan, Yidan Ma, Jianzhong Xu, Ying Chang, Shengli Guo

**Affiliations:** ^1^College of Educational Science, Bohai University, Jinzhou, China; ^2^Research Center of Brain and Cognitive Neuroscience, Liaoning Normal University, Dalian, China; ^3^Department of Counseling, Educational Psychology, and Foundations, College of Education, Mississippi State University, MS, United States

**Keywords:** homework, creativity, academic achievement, homework creativity, grade effect, homework behaviors

## Abstract

During the past several decades, the previous studies have been focusing on the related theoretical issues and measuring tool of homework behaviors (mainly including homework time, completion, and homework creativity). However, the effects of these homework behaviors on general creativity remain unknown. Employing a number of questionnaires, this study investigated two samples from middle schools of Mainland China. The results showed that (1) the eight-item version of Homework Creativity Behaviors Scale had acceptable validity and reliability; (2) compared with homework completion and homework time, homework creativity explained less variety of academic achievement (3.7% for homework creativity; 5.4% for completion and time); (3) homework creativity explained more variance of general creativity than that of homework completion and homework time accounted (7.0% for homework creativity; 1.3% for completion and time); and (4) homework creativity was negatively associated with grade level. Contrary to the popular beliefs, homework completion and homework creativity have positive effects on the students’ general creativity. Several issues that need further studies were also discussed.

## Introduction

Homework is an important part of the learning and instruction process. Each week, students around the world spend 3–14 hours on homework, with an average of 5 hours a week (Dettmers et al., 2009; OECD, 2014). The results of the previous studies and meta-analysis showed that the homework time is correlated significantly with students’ gains on the academic tests (Cooper et al., 2012; Fan et al., 2017; Fernández-Alonso et al., 2019).

Homework is a multi-faceted process which has many attributes – each attribute can be identified, defined, and measured independently (Guo and Fan, 2018). Some attributes, such as homework time (Núñez et al., 2013; Kalenkoski and Pabilonia, 2017), homework frequency (Fernández-Alonso et al., 2015), homework completion (Rosário et al., 2015), homework effort (Trautwein and Lüdtke, 2007; Fernández-Alonso et al., 2015), homework purpose (Trautwein and Lüdtke, 2009; Xu, 2010, 2021), homework performance and problems (Power et al., 2007), homework management behavior (Xu, 2008), homework expectation (Xu, 2017), and self-regulation of homework behavior (Yang and Tu, 2020), have been well recorded in the literature, and operationally defined and measured.

Recently, a research community has noticed the “creativity” in homework (in short form, “homework creativity”) who have raised some speculations about its effects on students’ academic achievement and general creativity disposition (Kaiipob, 1951; Beghetto and Kaufman, 2007; Kaufman and Beghetto, 2009; Guo, 2018; Guo and Fan, 2018; Chang, 2019). However, the scientific measurement of homework creativity has not been examined systematically. The relationship between homework creativity, academic achievement, and general creativity disposition, as well as the grade difference in homework creativity, are still in the state of conjectures consequently.

As a scientific probe to homework creativity, this study included three main sections. In the “Literature Review” section, the conceptualization and relevant measurement of homework creativity were summarized; the relationship between homework behaviors and academic achievements, general creativity, and the grade difference in homework behaviors and general creativity were also evaluated. These four main results related to the four research questions were also presented in the body of this article. They are reliability and validity of homework creativity behavior scale (HCBS), the relationships between the scores of HCBS and those of general creativity and academic achievement, and the grade effects of scores of HCBS. In the “Discussion” section, the scientific contributions and interpretations of the findings of this study were elaborated.

### Homework creativity

#### Conceptual background of homework creativity

As an attribute of homework process, homework creativity refers to the novelty and uniqueness of homework (Guo and Fan, 2018). Specifically, the ways relating to homework creativity with extant theoretical literature are presented below.

First, creativity is a natural part of homework process which serves as a sub-process of learning. Guilford (1950) is the first psychologist who linked creativity with learning, pointing out that the acquisition of creativity is a typical quality of human learning, and that a complete learning theory must take creativity into account.

Second, according to the Four-C Model of Creativity (e.g., Kaufman and Beghetto, 2009), the homework creativity can be divided mainly into the category of “Transformative Learning” (Mini-C creativity), which is different from the “Everyday Innovation” (Little-C creativity), “Professional Expertise” (Pro-C creativity), or “Eminent Accomplishments” (Big-C creativity, Beghetto and Kaufman, 2007; Kaufman and Beghetto, 2009; Kozbelt et al., 2011).

The Mini-C is defined as a type of intrapersonal creativity which has personal meaning, not solid contribution or breakthrough in a field (Beghetto and Kaufman, 2007, p. 76, Table 1). The most important point which distinguishes Mini-C from other types of creativity is the level of novelty of product. The Mini-C creativity involves the personal insight or interpretation which is new to a particular individual, but may be ordinary to others. The Little-C creativity refers to any small, but solid innovation in daily life. The Pro-C creativity is represented in the form of professional contribution which is still not a breakthrough. The Big-C creativity generates a real breakthrough appears in some field which is considered as something new to all human beings. The other difference is related with the subjects of sub-types of creativity. The Mini-C creativity mainly happens in all kinds of students. The Little-C creativity can be widely found in normal people. The Pro-C creativity’s masters are those who are proficient in some field. The Big-C creativity is related frequently with those giants who has made eminent contribution to human being.

**TABLE 1:** Basic information of samples 1 and 2 included.

	Sample 1					Sample 2				
		
	Grade 7	Grade 8	Grade 10	Grade 11	Total	Grade 7	Grade 8	Grade 10	Grade 11	Total
*N*	149	118	183	189	639	172	185	163	190	710
**Age**										
Mean/SD	13.29/0.63	13.89/0.79	15.96/0.58	17.02/0.56	15.27/1.64	13.33/0.70	14.29/0.65	16.17/0.61	16.44/0.83	15.06/1.47
Range	12–15	12–17	15–17	15–19	12–19	12–16	13–16	15–18	15–19	12–19
**Girls**										
Frequency	71	69	112	109	366	85	100	72	109	366
Percentage	51	58.5	61.2	57.7	57.2	49.4	54.1	44.2	57.4	51.5
**Homework Frequency**										
0 days	0	0	0	0	0	0	0	1	3	4
1–2 days	5	2	6	2	15	5	9	9	5	28
3 days	3	5	8	3	19	3	8	19	5	35
4 days	5	6	11	5	27	5	6	26	13	50
5 days	136	105	158	179	578	160	162	109	164	595

The Mini-C creativity frequently happens in learning process. When the contribution of the Mini-C creativity grows big enough, it can move into the category of the Little-C creativity, or the Big-C creativity. Most homework creativity is of Mini-C creativity, and of which a small part may grow as the Little-C and Big-C creativities. For example, when students independently find a unique solution to a problem in homework which has scientific meaning, a Little-C or Big-C occurs.

Third, the education researchers have observed homework creativity for many years and been manipulating them in educational practice. Kaiipob (1951) described that homework is a semi-guide learning process in which homework such as composition, report, public speech, difficult and complex exercises, experiments, and making tools and models consumes a lot of time and accelerate the development of students’ creativity disposition (p. 153).

In the recent years, creativity has become a curriculum or instruction goal in many countries (the case of United Kingdom, see Smith and Smith, 2010; Chinese case, see Pang and Plucker, 2012). Homework is the most important way that accomplish this goal. Considering Chinese in primary and secondary schools in China as an example, the curriculum standards have clearly required homework to cultivate students’ creative spirit, creative thinking, and ability to imagination since the year 2000. The results of Qian’s (2006) investigation revealed that the percent of these creative homework items in each unit fluctuates between 29 and 45%.

#### Previous instruments of homework behaviors

Those existent instruments measuring homework behavior can be divided into the following two categories: The single-indicator instruments and the multi-dimension instruments (Guo and Fan, 2018). The single-indicator instruments employ only one item to measure homework attributes, such as homework time (e.g., Trautwein and Lüdtke, 2007), homework frequency (e.g., De Jong et al., 2000), homework completion (e.g., Xu et al., 2019), and effort (e.g., Liu et al., 2013).

The typical multi-dimension instruments include Homework Process Inventory (Cooper et al., 1998), Homework Purpose Scale (Xu, 2010), Homework Performance Questionnaire (Pendergast et al., 2014), Homework Management Scale (HMS; Xu and Corno, 2003), Homework Evaluating Scale (Fernández-Alonso et al., 2015), Homework Problem Checklist (Anesko et al., 1987), Science Homework Scale (Tas et al., 2016), Homework Expectancy Value Scale (Yang and Xu, 2017), and Online Homework Distraction Scale (Xu et al., 2020).

Although the previous tools measured some dimensions of homework (Guo and Fan, 2018), there is hardly any tool that can be employed to gauge the homework creativity. Guo and Fan (2018) extracted several attributes (i.e., time, completion, quality, purpose, effort, creativity, sociality, liking) represented in the existent instruments of homework behaviors, and put forth a multi-faceted model of homework behaviors which intuitionally predicts the existence of homework creativity.

Under the guideline of the multi-faceted model (Guo and Fan, 2018), Guo (2018) developed a multi-dimensional homework behavior instrument, which detected the homework creativity as a dimension in the homework behavior of middle school students. A typical item of homework creativity in Guo (2018) is “The way I do my homework is different from others.” The subscale homework creativity reported by Guo (2018) needs to be improved because it has a small number of items with lower reliability.

Following Guo’s (2018) work, Chang (2019) conducted a new investigation focusing on homework creativity behavior. Using an open-ended questionnaire, a total of 30 students from primary, middle, and high schools were invited to answer this question, that is, “What characteristics can be considered as creative in the process of completing the homework?” Here, “creativity” refers to novelty, uniqueness, and high quality. A group of 23 specific behaviors were reported, among which the top 10 are as follows: Learning by analogy, open minded, one question with multiple solutions, unique solution, summarizing the cause of errors, constructing a personal understanding, analyzing knowledge points clearly, classifying homework contents, making more applications, having rich imagination, and a neat handwriting (see Chang, 2019, Table 4, p. 14). Based on these results of open-ended questionnaire, Chang (2019) invented a nine-item scale (see Table 1 and Supplementary Table S3 for details) called as the HCBS which has a good reliability coefficient (α = 0.87).

### Previous studies on the relationship between homework behaviors and academic achievement

In the literature, homework behaviors is one cluster of variables typically including homework time, homework completion, effort, purpose, frequency, etc. Academic achievement is an outcome of homework which is operationally measured using the scores on the standardized tests, or non-standardized tests (including final examinations, or teachers’ grades, or estimations by participants themselves, those forms were used widely in the literature, see Fan et al., 2017). Academic achievement may be affected by a lot of factors inherited in the process of learning (see Hattie, 2009 for an overview of its correlates). The relationship between homework behaviors and academic achievement is one of the most important questions in homework field, because it is related to the effectiveness of homework (Cooper et al., 2006, 2012; Fan et al., 2017).

Most of the previous studies focused on the relationship between homework time and academic achievement. Cooper et al. (2006) synthesized the primary studies published from 1989 to 2003, and found that the correlation between homework time of America students and their academic achievement was about 0.15. Fan et al. (2017) reviewed those individual studies published before June 2015, and reported that the averaged correlation between homework time of international students and their science, technology, engineering, and mathematics (STEM) academic achievement was about 0.20. Fernández-Alonso et al. (2017) investigated a representative sample of Spanish students (more than 26,000), and the results of multi-level analysis indicated that the correlation between homework time and academic achievement was negative at student level, but positive at school level (*r* = 0.16). Fernández-Alonso et al. (2019) took a survey on a big sample from 16 countries from Latin America, and reported that the relationship between homework time and academic achievement was very weak. Valle et al. (2019) analyzed the homework time, time management, and achievement of 968 Spain students finding that homework time management was positively related to academic achievement. Taken all these together, we will find that the homework has some small significant correlations with academic achievement, the average *r* = 0.15.

The correlation between homework completion and academic achievement has also been investigated for decades. Based on a review of 11 primary studies, Fan et al. (2017) reported a high correlation of 0.59 between them. Rosário et al. (2015) investigated 638 students, and demonstrated a correlation of 0.22 between amount of homework completed and math test scores. Xu et al. (2019) took a survey using a sample of 1,450 Chinese eighth graders, and found that the correlations between homework completion and the gains in math test scores ranged from 0.25 to 0.28. Dolean and Lervag (2022) employed the Randomized Controlled Trial design, and demonstrated that amount of homework completed has immediate effect on writing competency in which the effect of moderate amount of homework can last for 4 months. Integrating the aforementioned results, we can find that the averaged correlation between homework completion and academic achievement was higher than that between homework time with academic achievement.

Homework effort was also found to be correlated with academic achievement. Fan et al. (2017) reviewed four primary studies and returned that a medium correlation (*r* = 0.31) between homework effort and academic achievement. Two recent investigations showed that this relationship is positively and reciprocally related (*r* = 0.41–0.42) (Xu, 2020; Xu et al., 2021).

The effect of homework purpose was also correlated with the academic achievement. Fan et al. (2017) summarized four existent primary studies and reported an averaged correlation of 0.11 between them. Later, Rosário et al. (2015) found a similar correlation coefficient of these two variables on a sample of 638 students. Xu’s (2018) investigation revealed that the correlation between purpose and academic achievement was about 0.40. Sun et al. (2021) investigated a larger sample (*N* = 1,365), and found that the subscales of homework purpose had different correlation patterns with academic achievement (academic purpose is 0.40, self-regulatory purpose is 0.20, and approval-seeking purpose is 0.10).

Considering the case of homework creativity, there is only one study preliminarily investigated its relationship with academic achievement. Guo (2018) investigated a sample of 1,808 middle school students, and reported a significant correlation between homework creativity and academic achievement (*r* = 0.34, *p* < 0.05).

### Previous studies on the relationship between homework behaviors and general creativity

General creativity refers to the psychological attributes which can generate novel and valuable products (Kaufman and Glăveanu, 2019; Sternberg and Karami, 2022). These psychological attributes typically included attitude (e.g., willing to take appropriate risk), motivations (e.g., intrinsic motivation, curiosity), abilities (e.g., divergent thinking), and personality (e.g., independence) (Kaufman and Glăveanu, 2019; Long et al., 2022). These attributes can be assessed independently, or in the form of grouping (Plucker et al., 2019; Sternberg, 2019). For instance, the divergent thinking was measured independently (Kaufman et al., 2008). Also, the willing to take appropriate risk was measured in tools contain other variables (Williams, 1979). There are many studies examined the relationship between learning and general creativity in the past several decades indicating that the correlation between them was around 0.22 (e.g., Gajda et al., 2017; Karwowski et al., 2020).

Regarding the relationship between homework behaviors and general creativity, there are few studies which presented some contradictory viewpoints. Kaiipob (1951) posited that homework could accelerate development of students’ general creativity disposition, because the tasks in homework provide opportunities to exercise creativity. Cooper et al. (2012) argued that homework can diminish creativity. Furthermore, Zheng (2013) insisted that homework will reduce curiosity and the ability to challenging – the two core components of creativity. The preliminary results of Chang (2019) indicated that the score of HCBS is significantly correlated with scores of a test of general creativity, Williams’ creativity packet (*r* = 0.25–0.33, *p* < 0.05).

### Previous studies on the relationship between homework behaviors and homework creativity

In Guo and Fan’s (2018) theoretical work, homework creativity was combined from two independent words, homework and creativity, which was defined as a new attribute of homework process and was considered as a new member of homework behaviors. Up till now, there are two works providing preliminary probe to the relationship between homework behaviors and homework creativity. Guo (2018) investigated a sample of 1808 middle school students, and found that homework creativity was correlated significantly with liking (*r* = 0.33), correctness (*r* = 0.47), completion (*r* = 0.57), and purpose (*r* = 0.53). Based on another sample of Chinese students (elementary school students, *N* = 300; middle school students, *N* = 518; high school students, *N* = 386), Chang (2019) showed that the score of homework creativity was correlated significantly with homework time (*r* = 0.11), completion (*r* = 0.39), correctness (*r* = 0.63), effort (*r* = 0.73), social interaction (*r* = 0.35), quality (*r* = 0.69), interpersonal relation purpose (*r* = 0.17), and purpose of personal development (*r* = 0.41).

### Previous studies on grade differences of homework behaviors and general creativity

#### Grade differences of homework behaviors

As a useful indicator, homework time was recorded frequently (e.g., Cooper et al., 2006; Fan et al., 2017). A recent meta-analysis included 172 primary studies (total *N* = 144,416) published from 2003 to 2019, and demonstrated that time Chinese K-12 students spent on homework increased significantly along with increasing of grades (Zhai and Fan, 2021, October).

Regarding homework managing time, some studies reported the grade difference was insignificant. Xu (2006) surveyed 426 middle school students and found that there was no difference between middle school students and high school students. Xu and Corno (2003) reported that urban junior school students (*N* = 86) had no grade difference in homework Managing time. Yang and Tu (2020) surveyed 305 Chinese students in grades 7–9, and found that in managing time behavior, the grade differences were insignificant. The rest studies showed that the grade effect is significant. A survey by Xu et al. (2014) based on 1799 Chinese students in grades 10 and 11 showed that the higher level the grade, the lower level of time management.

#### Grade differences of general creativity

The findings from the previous studies suggested that the scores of general creativity deceases as the grade increases except for some dimensions. Kim (2011) reviewed the Torrance Tests of Creative thinking (TTCT) scores change using five datasets from 1974 to 2008, and reported that three dimensions of creative thinking (i.e., “Fluency,” “Originality,” and “Elaboration”) significantly decreased along with grades increase, while the rest dimension (i.e., “Abstractness of titles”) significantly increased when grades increase. Nie and Zheng (2005) investigated a sample of 3,729 participants from grades 3–12 using the Williams’ Creativity Assessment Packet (WCAP), and reported that the creativity scores decreased from grades 9–12. Said-Metwaly et al. (2021) synthesized 41 primary studies published in the past 60 years, and concluded that the ability of divergent thinking had a whole increase tendency from grades 1 to 12 with a decrease tendency from grades 8 to 11 at the same time.

### The purpose and questions of this study

What we have known about homework creativity hitherto is nothing except for its notation and a preliminary version of measurement. To get deeper understanding of homework creativity, this study made an endeavor to examine its relationships with relevant variables based on a confirmation of the reliability and validity of HCBS. Specifically, there are four interrelated research questions, as the following paragraphs (and their corresponding hypotheses) described.

(i) *What is the reliability and validity of the HCBS?*

Because the earlier version of the HCBS showed a good Cronbach α coefficient of 0.87, and a set of well-fitting indices (Chang, 2019), this study expected that the reliability and validity will also behave well in the current conditions as before. Then, we present the first set of hypotheses as follows:

**H1a:** The reliability coefficient will equal or greater than 0.80.

**H1b:** The one-factor model will also fit the current data well; and all indices will reach or over the criteria as the expertise suggested.

(ii) *What degree is the score of the HCBS related with academic achievement?*

As suggested by the review section, the correlations between homework behaviors and academic achievement ranged from 0.15 and 0.59 (e.g., Fan et al., 2017), then we expected that the relationship between homework creativity and academic achievement will fall into this range, because homework creativity is a member of homework behaviors.

The results of the previous studies also demonstrated that the correlation between general creativity and academic achievement changed in a range of 0.19–0.24 with a mean of 0.19 (Gajda et al., 2017). Because it can be treated as a sub-category of general creativity, we predicted that homework creativity will have a similar behavior under the current condition.

Taken aforementioned information together, Hypothesis H2 is presented as follows:

**H2:** There will be a significant correlation between homework creativity and academic achievement which might fall into the interval of 0.15–0.59.

(iii) *What degree is the relationship between HCBS and general creativity?*

As discussed in the previous section, there are no inconsistent findings about the relationship between the score of HCBS and general creativity. Some studies postulated that these two variables be positive correlated (e.g., Kaiipob, 1951; Chang, 2019); other studies argued that this relationship be negative (e.g., Cooper et al., 2012; Zheng, 2013). Because homework creativity is a sub-category of general creativity, we expected that this relationship would be positive and its value might be equal or less than 0.33. Based on those reasoning, we presented our third hypothesis as follows:

**H3:** The correlation between homework creativity and general creativity would be equal or less than 0.33.

(iv) *What effect does grade have on the HCBS score?*

Concerning the grade effect of homework behaviors, the previous findings were contradictory (Xu et al., 2014; Zhai and Fan, 2021, October). However, the general creativity decreased as the level of grade increases from grade 8 to grade 11 (Kim, 2011; Said-Metwaly et al., 2021). Taken these previous findings and the fact that repetitive exercises increase when grades go up (Zheng, 2013), we were inclined to expect that the level of homework creativity is negative correlated with the level of grade. Thus, we presented our fourth hypothesis as follows:

**H4:** The score of HCBS might decrease as the level of grades goes up.

## Materials and methods

### Participants

#### Settings

To get more robust result, this study investigated two convenient samples from six public schools in a medium-sized city in China. Among them, two schools were of high schools (including a key school and a non-key school), and the rest four schools were middle schools (one is key school, and the rest is non-key school). All these schools included here did not have free lunch system and written homework policy. Considering the students were mainly prepared for entrance examination of higher stage, the grades 9 and 12 were excluded in this survey. Consequently, students of grades 7, 8, 10, and 11 were included in our survey. After getting permission of the education bureau of the city investigated, the headmasters administrated the questions in October 2018 (sample 1) and November 2019 (sample 2).

#### Sample 1

A total of 850 questionnaires were released and the valid number of questionnaires returned is 639 with a valid return rate of 75.18%. Therefore, there were 639 valid participants in sample 1. Among them, there were 273 boys and 366 girls (57.2%); 149 participants from grade 7 (23.31%), 118 from grade 8 (18.47%), 183 from grade 10 (28.64%), and 189 from grade 11 (29.58%); the average age was 15.25 years, with a standard deviation (SD) of 1.73 years. See Table 1 for the information about each grade.

Those participants included received homework assignments every day (see Table 1 for the distribution of homework frequency). During the working days, the averaged homework time was 128.29 minutes with *SD* = 6.65 minutes. In the weekend, the average homework time was 3.75 hours, with *SD* = 0.22 hours. The percentage distribution here is similar with that of a national representative sample (Sun et al., 2020), because the values of Chi-squared (χ^2^) were 7.46 (father) and 8.46 (mother), all *p*-values were above 0.12 (see Supplementary Table S1 for details).

#### Sample 2

Another package of 850 questionnaires were released. The valid number of questionnaires returned is 710 with a valid return rate of 83.53%. Among them, there were 366 girls (51.50%); 171 participants from grade 7 (24.23%), 211 from grade 8 (26.06%), 190 from the grade 10 (22.96%), and 216 from grade 11 (26.76%); the average age was 15.06 years, with *SD* = 1.47 years.

Those participants included received homework assignments almost each day (see Table 1 for details for the distribution of homework frequency). During the working days, the averaged homework time was 123.02 minutes with *SD* = 6.13 minutes. In weekend, the average homework time was 3.47 hours, with *SD* = 0.21 hours.

The percentage distribution here is insignificantly different from that of a national representative sample (Sun et al., 2020), because the values of χ^2^ were 5.20 (father) and 6.05 (mother), *p*-values were above 0.30 (see Supplementary Table S1 for details).

### Instruments

#### The homework creativity behavior scale

**FIGURE 1:**
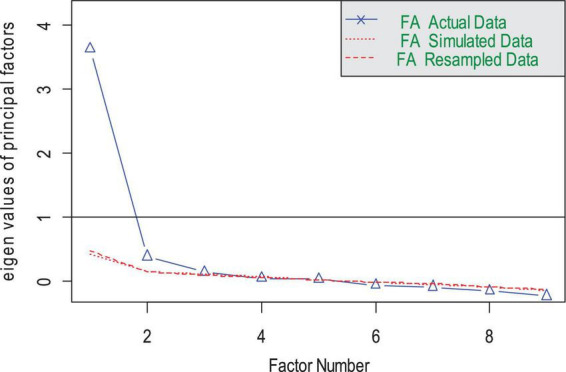
Parallel analysis scree plots of the HCBS data.

**FIGURE 2:**
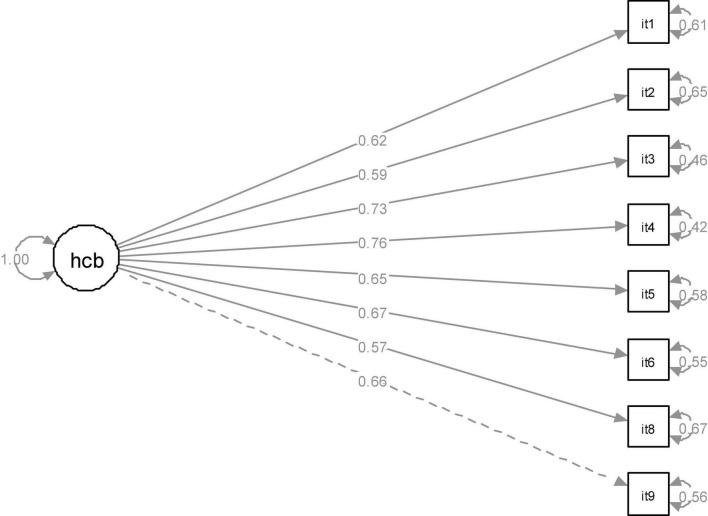
The standardized solution for HCBS eight-item model. hcb, homework creativity behavior; it 1∼9, item1 ∼6, 8∼9.

The HCBS contains nine items representing students’ creativity behaviors in the process of completing homework (for example, “I do my homework in an innovative way”) (Chang, 2019, see Supplementary Table S3 for details). The HCBS employs a 5-point rating scale, where 1 means “completely disagree” and 5 means “completely agree.” The higher the score, the stronger the homework creative behavior students have. The reliability and validity of the HCBS can be found in Section “Reliability and validity of the homework creativity behavior scale” (see Table 2 and Figures 1, 2 for details).

**TABLE 2:** Results of item discrimination analysis and exploratory factor analysis.

Items	Item-scale correlations	Factor loading	Communality
1. I do my homework in an innovative way	0.70**^a^ (0.67**^b^)	0.66	0.44
2. I do my homework without sticking to what I have learned in class	0.65** (0.63**)	0.62	0.38
3. I found a better solution to complete homework	0.75** (0.74**)	0.76	0.58
4. I use a simpler method to do the homework	0.74** (0.75**)	0.75	0.56
5. My rich imagination can be reflected in my homework	0.67** (0.70**)	0.62	0.38
6. I designed new problems on the basis of teachers	0.69** (0.74**)	0.63	0.40
7. I designed a neat, clean and clear homework format by myself^c^	0.54** (0.74**)	0.40	0.16
8. I have my own unique insights into homework	0.67** (0.68**)	0.57	0.33
9. I give multiple solutions to a problem	0.70** (0.73**)	0.63	0.39
KMO		0.89	
Eigenvalue		3.63	
Proportion of variance explained		0.40	

**p < 0.01, two side-tailed. The same for below.

^a^Correlations for sample 1; ^b^Correlations for sample 2. ^c^Seventh item should be removed away according to the results of CFA (see section “Reliability and validity of the HCBS” for details).

#### Homework management scale

The HMS contains 22 items describing specific behaviors related to self-management in homework (for example, “I will choose a quiet place to do my homework” or “Tell myself to calm down when encountering difficulties”) (Xu and Corno, 2003; Xu, 2008). The HMS employs a 5-point Likert scale, ranging from 1 (completely disagree) to 5 (completely agree). All items can be divided into five dimensions, i.e., arranging environment, managing time, focusing attention, monitoring motivation, and monitoring and controlling emotion. Among them, the monitoring and controlling emotion dimension adopts a method of reverse scoring.

Except for the internal consistency of arranging environment in sample 1, which is 0.63, the internal consistency coefficients of the five dimensions based two samples in this study are all greater than 0.7, ranging from 0.70 to 0.79. The Cronbach’s coefficients of the overall HMS-based two samples are 0.88 and 0.87, respectively. The ω coefficients of the dimensions of HMS ranged from 0.64 to 0.80. The ω coefficients of the HMS total scores were 0.88 and 0.87 for samples 1 and 2, respectively. Those reliability coefficients were acceptable for research purpose (Clark and Watson, 1995; Peterson and Kim, 2013).

#### Williams’ creativity assessment packet

The WCAP including a total of 40 items is a revised version to measure general disposition of creativity (for example, “I like to ask some questions out of other’s expectation” or “I like to imagine something novel, even if it looks useless”) (Williams, 1979; Wang and Lin, 1986; Liu et al., 2016). The WCAP uses a 3-point Likert scales, in which 1 = disagree, 2 = uncertain, and 3 = agree. The higher WCAP score, the higher is the general creativity level. All items of WCAP can be scattered into four dimensions: adventure, curiosity, imagination, and challenge (Williams, 1979; Wang and Lin, 1986; Liu et al., 2016). In this study, the Cronbach’s α coefficients of adventure, curiosity, imagination, challenge, and total scale are 0.62, 0.71, 0.78, 0.64, and 0.90, respectively. The ω coefficients were in sequence 0.61, 0.70, 0.77, 0.63, and 0.90 for adventure, curiosity, imagination, challenge, and the total score of WCAP. The correlations between the four dimensions of WCAP are between 0.47 and 0.65. The patterns of reliability coefficients and correlations between dimensions are similar to those results reported by the previous studies (Williams, 1979; Wang and Lin, 1986; Liu et al., 2016) which stand acceptable reliability and validity (Clark and Watson, 1995; Peterson and Kim, 2013).

#### Homework indicators

##### Homework time

The participants were asked to report the time spent on homework in the past week. This technique has been employed widely in many international survey programs, such as PISA from OECD (e.g., Trautwein and Lüdtke, 2007). The items are as follows: (1) “Every day, from Monday to Friday, in last week, how many minutes you spent on homework?” The options are as follows: (A) 0–30 min; (B) 31–60 min (C) 61–90 min (D) 91–120 min; (E) 121–180 min; (F) 181 min or more. (2) “In last weekend, how many hours you spent on homework?” The options are as follows: (A) 0–1 h; (B) 1.1–3 h; (C) 3.1–5 h; (D) 5.1–7 h; (E) 7.1 h or more.

##### Homework completion

The homework completion is a useful indicator demonstrated in the previous studies (Welch et al., 1986; Austin, 1988; Swank, 1999; Pelletier, 2005; Wilson, 2010), and had large correlation with achievement, as a meta-analytic results suggested (Fan et al., 2017). In the survey of this study, the participants were also asked to estimate a percent of the completion of homework in the past week and fill in the given blank space. It includes three items which are as follows: “What is the percentage of Chinese/Maths/English homework assignment you completed in the last week?” “Please estimate and write a number from 0 to 100 in the blank space.”

#### Academic achievement

To record the academic achievement, an item required participants to make a choice based on their real scores of tests, not estimate their tests scores. The item is, “In the last examination, what is the rank of your score in your grade?” (A) The first 2%; (B) The first 3–13%; (C) The first 14–50%; (D) The first 51–84%; (E) The last 16%. The options here correspond to the percentage in the normal distribution, it is convenient to compute a *Z*-score for each student.

The method employed here is effective to retrieve participants’ test scores. First, the self-report method is more effective than other method under the condition of anonymous investigation. To our knowledge, participants do not have the will to provide their real information in the real name format. Second, this method transforms test scores from different sources into the same space of norm distribution which benefits the comparisons. Third, the validity of this method has been supported by empirical data. Using another sample (*N* = 234), we got the academic achievement they reported and real test scores their teacher recorded. The correlation between ranks self-reported and the real scores from Chinese test were *r* = 0.81, *p* < 0.001; and the correlation coefficient for mathematics was also large, i.e., *r* = 0.79, *p* < 0.001.

### Data collection procedure

There are three phases in data collection. The first one is the design stage. At this stage, the corresponding author of this study designed the study content, prepared the survey tools, and got the ethical approve of this project authorized from research ethic committee of school the corresponding author belongs to.

The second stage is to releasing questionnaire prepared. The questionnaire was distributed and retrieved by the head master of those classes involved. Neither the teachers nor the students knew the purpose of this research. During this stage, students can stop answering at any time, or simply withdraw from the survey. None of the teachers and students in this study received payment.

The third stage is the data entry stage. At this stage, the corresponding author of this study recruited five volunteers majored in psychology and education, and explained to them the coding rules, missing value processing methods, identification of invalid questionnaires, and illustrated how to deal with these issues. The volunteers used the same data template for data entry. The corresponding author of this study controlled the data entry quality by selective check randomly.

### Data analysis strategies

#### R packages employed

The “psych” package in R environment (R Core Team, 2019) was employed to do descriptive statistics, correlation analysis, mean difference comparisons, exploratory factor analysis (EFA), reliability Analysis (Revelle, 2022); and the “lavaan” package was used in confirmatory factor analysis (CFA) and measurement invariance test (Rosseel, 2012); and the “semPlot” package was employed to draw the picture of CFA’s outputs (Epskamp et al., 2022).

#### Analysis strategies of exploratory factor analysis and reliability

Sample 1 was used for item analysis, EFA, reliability analysis. In EFA, factors were extracted using maximum likelihood, and the promax method served as the rotation method. The number of factors were determined according to the combination of the results from screen plot, and the rule of Eigenvalues exceeding 1.0, and parallel analysis (Luo et al., 2019).

The Cronbach’s α and MacDonald’s ω test were employed to test the reliability of the scale. The rigorous criteria that α ≥ 0.70 (Nunnally and Bernstein, 1994) and ω ≥ 0.7 (Green and Yang, 2015) were taken as acceptable level of the reliability of HCBS.

#### Analysis strategies of confirmatory factor analysis

As suggested by Hu and Bentler (1999), two absolute goodness-of-fit indices, namely, the root mean square error of approximation (RMSEA) and the standardized root mean square residual (SRMR), and two relative goodness-of-fit indices, namely, comparative fit index (CFI) and Tucker–Lewis Index (TLI) were recruited as fitting indicators. The absolute goodness-of-fit indices are less than 0.08, and the relative goodness-of-fit indices greater than 0.90 are considered as a good fit. The CFA was conducted using the second sample.

#### Strategies for measurement invariance

Measurement invariance testing included four models, they are Configural invariance (Model 1), which is to test whether the composition of latent variables between different groups is the same; Weak invariance (Factor loading invariance, Model 2), which is to test whether the factor loading is equal among the groups; Intercept invariance (Model 3), that is, whether the intercepts of the observed variables are equal; Strict equivalent (Residual Variance invariance, Model 4), that is, to test whether the error variances between different groups are equal (Chen, 2007; Putnick and Bornstein, 2016).

Since the χ^2^ test will be affected easily by the sample size, even small differences will result in significant differences as the sample size will increase. Therefore, this study used the changes of model fitting index CFI, RMSEA, and SRMR (ΔCFI, ΔRMSEA, and ΔSRMR) to evaluate the invariance of the measurement. When ΔCFI ≤ 0.010, ΔRMSEA ≤ 0.015, and ΔSRMR ≤ 0.030 (for metric invariance) or 0.015 (for scalar or residual invariance), the invariance model is considered acceptable (Cheung and Rensvold, 2002; Chen, 2007; Putnick and Bornstein, 2016).

#### Strategies of controlling common methods biases

The strategy of controlling common methods biases is mainly hided in the directions. Each part of the printed questionnaire had a sub-direction which invites participants answer the printed questions honestly. The answer formats between any two neighboring parts were different from each other which requested participants change their mind in time. For example, on some part, the answering continuum varied from “1 = totally disagreed” to “5 = total agreed,” while the answering continuum on the neighboring part is the from “5 = totally disagreed” to “1 = total agreed.” Additionally, according to the suggestion of the previous studies, the one factor CFA model and the bi-factor model can be used to detect the common methods biases (e.g., Podsakoff et al., 2012).

## Results

### Detection of common method biases

The fitting results of the one-common-factor model using CFA technique were as follows: χ^2^ = 15,073, *df* = 3320, *p* < 0.001; χ^2^/*df* = 4.54, CFI = 0.323, TLI = 0.306, RMSEA = 0.071, 90% CI: 0.070–0.072, and SRMR = 0.101. The results of the bi-factor model under CFA framework were presented as follows: χ^2^ = 2,225.826, *df* = 117, *p* < 0.001; χ^2^/*df* = 19.024, CFI = 0.650, TLI = 0.543, RMSEA = 0.159, 90% CI: 0.154–0.164, and SRMR = 0.127. These poor indices of the two models suggested that the one-common-factor model failed to fit the data well and that the biases of common method be ignored (Podsakoff et al., 2012).

### Reliability and validity of the homework creativity behavior scale

#### Item analysis

Based on the sample 1, the correlation coefficients between the items of the HCBS were between 0.34 and 0.64, *p*-values were below 0.01. The correlations between the items and the total score of HCBS vary from 0.54 to 0.75 (*p*-values are below 0.01). On the condition of sample 2, the correlations between the items fluctuate between 0.31 and 0.58, the correlation coefficients between the items and the total score of the HCBS change from 0.63 to 0.75 (*p*-values were below 0.01). All correlation coefficients between items and total score are larger than those between items and reached the criterion suggested (Ferketich, 1991; see Table 2 for details).

#### Results of exploratory factor analysis

The EFA results (based on sample 1) showed that the KMO was 0.89, and the χ^2^ of Bartlett’s test = 1,666.07, *p* < 0.01. The rules combining eigenvalue larger than 1 and the results of parallel analysis (see Figure 1 for details) suggested that one factor should be extracted. The eigenvalue of the factor extracted was 3.63. The average variance extracted was 0.40. This factor accounts 40% variance with factor loadings fluctuating from 0.40 to 0.76 (see Table 2).

#### Results of confirmatory factor analysis

In the CFA situation (based on sample 2) the fitting indices of the nine-item model of the HCBS are acceptable marginally, they are χ^2^ = 266.141; *df* = 27; χ^2^/*df* = 9.857; CFI = 0.904; TLI = 0.872; RMSEA = 0.112; 90% CI: 0.100–0.124; SRMR = 0.053.

The modification indices of item 7 were too big (MI value = 74.339, *p* < 0.01), so it is necessary to consider to delete item 7. Considering its content of “I designed a neat, clean and clear homework format by myself,” item 7 is an indicator of strictness which is weakly linked with creativity. Therefore, the item 7 should be deleted.

After removing item 7, the fitting results were, χ^2^ = 106.111; *df* = 20; χ^2^/*df* = 5.306; CFI = 0.957; TLI = 0.939; RMSEA = 0.078; 90% CI: 0.064–0.093; SRMR = 0.038). The changes of the fitting indices of the two nested models (eight-item *vs.* nine-item models) are presented as follows: Δχ^2^ = 160.03, Δ*df* = 7, χ^2^ (α = 0.01, *df* = 7) = 18.48, *p* < 0.05. After deleting item 7, both CFI and TLI indices increased to above 0.93, and RMSEAs decreased below 0.08 which suggested that the factor model on which eight items loaded fitted the data well. The average variance extracted was 0.50 which is adequate according to the criteria suggested by Fornell and Larcker (1981). The standardized solution for the eight-item model of the HCBS was shown in Figure 2.

#### Correlations between the homework creativity behavior scale and similar concepts

The results showed that the score of the HCBS was significantly correlated with the total score and four dimensions of WCAP and their correlation coefficients ranged from 0.20 to 0.29, *p*-values were below 0.01. Similarly, the correlations between the score of the HCBS and the scores of arranging environment, managing time, motivation management, and controlling emotion, and total score of the HMS ranged from 0.08 to 0.22, *p*-values were 0.01; at the meanwhile, the correlation between the score of HCBS and the distraction dimension of the HMS was *r* = –0.14, *p*-values were 0.01. The HCBS score was also significantly related to homework completion (*r* = 0.18, *p* < 0.01), but insignificantly related to homework time (see Table 3 for details).

**TABLE 3:** Correlation matrix between variables included and the corresponding descriptive statistics.

	1	2	3	4	5	6	7	8	9	10	11	12	13	14	15	16	17
(1) Grade^a^	1	0.00	0.00	–0.40**	0.00	–0.02	–0.06	–0.06	–0.06	0.20**	–0.11**	–0.15**	–0.13**	–0.06	–0.06	–0.25**	0.00
(2) TWk^b^	0.00	1	0.46**	0.09*	0.04	0.02	0.05	0.04	0.03	–0.06	0.02	0.02	0.01	0.01	0.01	0.02	0.01
(3) TWw^b^	0.00	0.39**	1	0.19**	0.02	0.06	0.07	0.01	0.05	–0.03	0.01	0.03	0.00	0.02	0.04	0.02	0.08*
(4) HCp^b^	–0.25**	0.15**	0.14**	1	0.19*	0.20**	0.18**	0.18**	0.21**	–0.08*	0.10*	0.09*	0.08	0.06	0.14**	0.18**	0.26**
(5) HMSt	0.04	0.09	0.08	0.19*	1	0.81**	0.85**	0.83**	0.86**	–0.29*	0.21**	0.22**	0.19**	0.11	0.26**	0.11	0.16**
(6) AE^b^	–0.02	0.07	0.13**	0.15**	0.76**	1	0.74**	0.57**	0.69**	–0.02	0.08*	0.10*	0.07	0.01	0.14	0.08*	0.15**
(7) MT^b^	0.02	0.08*	0.11**	0.21**	0.83**	0.70**	1	0.67**	0.74**	–0.01	0.18**	0.18**	0.15**	0.08	0.22**	0.10*	0.17**
(8) MM^b^	0.01	0.08*	0.03	0.21**	0.85**	0.55**	0.65**	1	0.71**	0.05	0.20**	0.24**	0.15**	0.11**	0.22**	0.22**	0.14**
(9) CE^b^	0.03	0.05	0.04	0.22**	0.85**	0.61**	0.70**	0.75**	1	0.02	0.17**	0.20**	0.15*	0.06	0.22**	0.13**	0.14**
(10) FA^b^	0.06	0.01	0.01	–0.14**	–0.18*	–0.14**	–0.13**	–0.01	–0.12**	1	0.17	0.06*	0.17**	0.23**	0.09**	–0.14**	0.00
(11) WCAPt^b,c^											1	0.84**	0.88**	0.87**	0.84**	0.29**	0.09*
(12) AD^b,c^												1	0.67**	0.61**	0.68**	0.29**	0.07
(13) CU^b,c^													1	0.67**	0.66**	0.26**	0.08*
(14) IM^b,c^														1	0.62**	0.20**	0.04
(15) CH^b,c^															1	0.28**	0.16**
(16) HCb^b,c^	–0.21**	0.02	–0.04	0.20**	0.22*	0.18**	0.20**	0.27**	0.24**	–0.13**						1	0.24**
(17) AA^b,c^	0.00	–0.07	0.02	0.23**	0.22*	0.24**	0.23**	0.20**	0.24**	–0.15**						0.26**	1
*M*	–	2.84/2.66	4.36/4.06	0.89/.87	3.48/.32	3.77/3.52	3.74/3.45	3.48/3.27	3.76/3.60	2.67/2.77	/3.19	/2.36	/2.34	/2.30	/2.43	3.24/3.19	0/0
*SD*	–	0.98/0.92	1.26/1.33	0.14/0.16	0.61/0.69	0.75/0.89	0.89/0.93	0.97/1.01	0.90/0.94	0.90/0.98	/0.30	/0.33	/0.34	/0.40	/0.31	0.82/0.84	1/1
α^d^					0.88/0.87	0.63/0.71	0.77/0.70	0.76/0.74	0.76/0.79	0.78/0.76	/0.89	/0.61	/0.70	/0.75	/0.64	0.86/0.86	
Ω					0.88/0.87	0.64/0.71	0.77/0.71	0.76/0.74	0.76/0.79	0.80/0.78	/0.90	/0.61	/0.70	/0.77	/0.63		

About correlation between variables, the results of sample 1 and sample 2 were presented in the lower, upper triangle, respectively.

^a^In analyses, grades 7, 8, 10, and 11 were valued 1, 2, 3, and 4, respectively.

^b^TWk, the time spent on homework in the weekend; TWw, the time spent on homework from Monday to Friday; HCp, homework completion; HMSt, total score of homework management scale; AE, arrange environment; MT, manage time; MM, monitor motivation; CE, control emotion; FA, focus attention; WCAPt, WCAP total score; AD, adventure; CU, curiosity; IM, imagination; CH, challenging; HCb, homework creativity behavior; AA, academic achievement.

^c^Since sample 1 did not answer the WCAP, so the corresponding cells in the lower triangle are blank. *p < 0.05, two side-tailed, the same for below.

^d^Since there is only one item from variable 1 to 4, the α and ω coefficients cannot be computed.

**TABLE 4:** Regression analyses of homework creative behavior on academic achievement and general creativity.

Steps	Predictors	Dependent variables					
		
		AA	WCAPt	Adventure	Curiosity	Imagination	Challenge
Step 1	Gender	–0.087*	–0.041	–0.006	–0.067	0.015	0.015
	Grade	0.002	–0.106**	–0.130**	–0.139**	–0.057	–0.056
	Adjusted *R*^2^	0.008	0.013	0.017	0.024	0.003	0.003
	*F*	2.685	4.738*	6.103**	8.82**	1.197	1.197
Step 2	TWk	0.059	–0.033	–0.068	–0.027	–0.005	–0.019
	TWw	–0.045	0.022	–0.037	0.018	0.013	0.002
	HCp	0.250**	0.123**	0.123**	0.111*	0.053	0.148**
	Adjusted *R*^2^	0.066	0.026	0.031	0.035	0.006	0.026
	ΔAdjusted *R*^2^	0.054	0.013	0.016	0.011	0.003	0.023
	*F*	9.906**	3.745**	4.528**	5.05**	0.836	3.772**
Step 3	HCb	0.206**	0.284**	0.272**	0.243**	0.225**	0.236**
	Adjusted *R*^2^	0.103	0.096	0.095	0.086	0.050	0.075
	ΔAdjusted *R*^2^	0.037	0.070	0.064	0.051	0.044	0.049
	*F*	13.41**	12.5**	12.37**	11.02**	6.168**	9.471**

AA, academic achievement; WCAPt, total score of WCAP; TWk, time spent on homework in week days; TWw, time spent on homework in weekend; HCp, homework completion; HCb, homework creativity behavior.

#### Correlations between the homework creativity behavior scale and distinct concepts

The correlation analysis results demonstrated that both the correlation coefficients between the score of HCBS and the time spent on homework in week days, and time spent on in weekend days were insignificant (*r*-values = 0.02, *p*-values were above 0.05), which indicated a non-overlap between two distinct constructs of homework creativity and time spent on homework.

#### Reliability analyses

The results revealed that both the Cronbach’s α coefficients of sample 1 and sample 2 were 0.86, which were greater than a 0.70 criteria the previous studies suggest (Nunnally and Bernstein, 1994; Green and Yang, 2015).

### Effect of homework creativity on academic achievement

The results (see Table 4) of hierarchical regression analyses demonstrated that (1) gender and grade explained 0.8% variation of the score of academic achievement. This number means closing to zero because the regression equation failed to pass the significance test; (2) homework time and completion explained 5.4% variation of academic achievement; considering the β coefficients of the time spent on homework is insignificant, this contribution should be attributed to homework completion totally, and (3) the score of the HCBS explained 3.7% variation of the academic achievement independently.

### Effect of homework creativity on general creativity

The results showed the following (see Table 4 for details):

(1) Gender and grade explained 1.3% variation of the total score of general creativity (i.e., the total score of WACP); homework time and completion explained 1.3% variation of the total score of general creativity disposition; and the score of the HCBS independently explained 7.0% variation of the total score of general creativity.

(2) Gender and grade explained 1.7% variation of the adventure score, and homework time and completion explained 1.6% variation of the adventure score, and the score of the HCBS independently explained 6.4% variation of the adventure score.

(3) Gender and grade explained 2.4% variation of the curiosity score, and homework time and completion explained 1.1% variation of the curiosity score, and the score of the HCBS independently explained 5.1% variation of the curiosity score.

(4) Gender and grade explained 0.3% variation of the imagination score, homework time completion explained 0.3% variation of the imagination score. The real values of the two “0.3%” are zeros because both the regression equations and coefficients failed to pass the significance tests. Then the score of the HCBS independently explained 4.4% variation of the imagination score.

(5) Gender and grade explained 0.3% variation of the score of the challenge dimension, homework time and completion explained 2.3% variation of the challenge score, and the score of the HCBS independently explained 4.9% variation of the challenge score.

### Grade differences of the homework creativity behavior scale

#### Test of measurement invariance

The results of measurement invariance test across four grades indicated the following:

(1) The fitting states of the four models (Configural invariance, Factor loading invariance, Intercept invariance, and Residual variance invariance) were marginally acceptable, because values of CFIs (ranged from 0.89 to 0.93), TLIs (varied from 0.91 to 0.93), RMSEAs (fluctuated from 0.084 to 0.095), and SRMRs (changed from 0.043 to 0.074) located the cutoff intervals suggested by methodologists (Cheung and Rensvold, 2002; Chen, 2007; Putnick and Bornstein, 2016; see Table 5 for fitting indices, and refer to Supplementary Table S2 for the estimation of parameters).

**TABLE 5:** Fitting results of invariance tests across grades.

Invariance models	χ^2^	*df*	χ^2^/*df*	RMSEA	90% CI	SRMR	CFI	TLI	Model comparison	ΔCFA	ΔRMSEA	ΔSRMR
1. Configural	321.737	80	4.02	0.095	0.084–0.106	0.043	0.934	0.908				
2. Factor loading	363.219	101	3.60	0.088	0.078–0.098	0.059	0.928	0.921	2 *vs.* 1	–0.006	–0.007	0.016
3. Intercept	414.701	122	3.40	0.084	0.076–0.094	0.064	0.920	0.927	3 *vs.* 2	–0.008	–0.004	0.005
4. Residual variances	539.345	146	3.69	0.089	0.081–0.098	0.074	0.893	0.918	4 *vs.* 3	–0.027	0.005	0.010

(2) When setting factor loadings equal across four grades (i.e., grades 7, 8, 10, and 11), the ΔCFA was –0.006, ΔRMSEA was –0.007, and ΔSRMR was 0.016 which indicated that it passed the test of factor loading invariance. After adding the limit of intercepts equal across four groups, the ΔCFA was –0.008, ΔRMSEA was –0.004, and the ΔSRMR was 0.005 which supported that it passed the test of intercept invariance. At the last step, the error variances were also added as equal, the ΔCFA was –0.027, ΔRMSEA was 0.005, and the ΔSRMR was 0.019 which failed to pass the test of residual variance invariance (see Table 5 for changes of fitting indices). Taking into these fitting indices into account, the subsequent comparisons between the means of factors can be conducted because the residuals are not part of the latent factor (Cheung and Rensvold, 2002; Chen, 2007; Putnick and Bornstein, 2016).

#### Grade differences in homework creativity and general creativity

**FIGURE 3:**
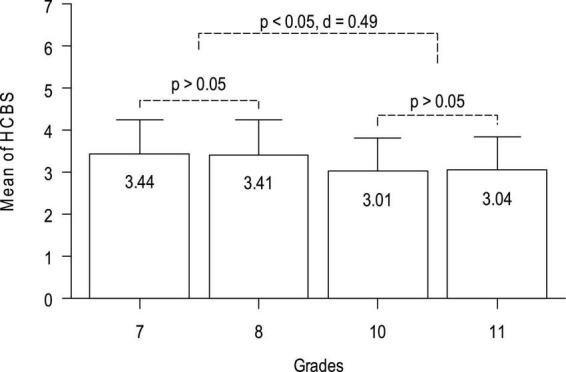
The mean differences of the HCBS between the groups of grades.

The results of ANOVA showed that there were significant differences in the HCBS among the four grades [*F*(3,1345) = 27.49, *p* < 0.001, η^2^ = 0.058, see Table 6 for details]. Further post-test tests returned that the scores of middle school students were significantly higher than those of high school students (Cohen’s *d* values ranged from 0.46 to 0.54; the averaged Cohen’s *d* = 0.494), and no significant difference occurs between grades 7 and 8, or between grades 10 and 11. See Figure 3 for details.

**TABLE 6:** Grade differences in HCBS.

	*N*	Mean	SD	Skewness	Kurtosis	*F*
Grade 7	321	3.44	0.81	–0.28	–0.29	27.49***
Grade 8	303	3.41	0.83	0.06	–0.77	
Grade 10	346	3.01	0.80	0.13	–0.08	
Grade 11	379	3.04	0.80	0.25	–0.31	

***p < 0.001.

## Discussion

To address the gap in the previous research on homework creativity, this study examined the psychometric proprieties of the HCBS and its relationship with academic achievement and general creativity. The main findings were (1) Hypotheses H1a and H1b were supported that the reliability and validity of the HCBS were acceptable; (2) Hypothesis H2 was supported that the correlation between the score of the HCBS and academic achievement was significant (*r*-values = 0.23–0.26 for two samples); (3) Hypothesis H3 received support that the correlation between the scores of HCBS and WCAP was significant (*r*-values = 0.20–0.29 for two samples); and (4) the H4 was supported from the current data that the score of high school students’ was lower than that of the middle school students’ (Cohen’s *d* = 0.49).

### The positive correlations among homework creativity, homework completion, and general creativity

The first key finding should be noted is that the positive correlations with between pairs of homework creativity, homework completion, and general creativity. This result is inconsistent with prediction of an argument that homework diminishes creativity (Cooper et al., 2012; Zheng, 2013). Specifically, the correlation between homework completion and curiosity was insignificant (*r* = 0.08, *p* > 0.05) which did not support the argument that homework hurts curiosity of creativity (Zheng, 2013). The possible reason may be homework can provide opportunities to foster some components of creativity by independently finding and developing new ways of understanding what students have learned in class, as Kaiipob (1951) argued. It may be the homework creativity that served as the way to practice the components of general creativity. In fact, the content of items of the HCBS are highly related with creative thinking (refer to Table 2 for details).

### Possible reasons of the grade effect of the score of the homework creativity behavior scale

The second key finding should be noted is that the score of the HCBS decreased as the level of grades increased from 7 to 11. This is consistent with the basic trend recorded in the previous meta-analyses (Kim, 2011; Said-Metwaly et al., 2021). There are three possible explanations leading to this grade effect. The first one is the repetitive exercises in homework. As Zheng (2013) observed, to get higher scores in the highly competitive entrance examination of high school and college, those Chinese students chose to practice a lot of repetitive exercises. The results of some behavior experiments suggested that repetitive activity could reduce the diverse thinking of subjects’ (e.g., Main et al., 2020). Furthermore, the repetitive exercises would lead to fast habituation (can be observed by skin conductance records) which hurts the creative thinking of participants (Martindale et al., 1996). The second explanation is that the stress level in Chinese high schools is higher than in middle school because of the college entrance examination. The previous studies (e.g., Beversdorf, 2018) indicated that the high level of stress will trigger the increase activity of the noradrenergic system and the hypothalamic–pituitary–adrenal (HPA) axis which could debase the individual’s performance of creativity. Another likely explanation is the degree of the certainty of the college entrance examination. The level of certainty highly increases (success or failure) when time comes closer to the deadline of the entrance examination. The increase of degree of certainty will lead to the decrease of activity of the brain areas related to curiosity (e.g., Jepma et al., 2012).

### The theoretical implications

From the theoretical perspective, there are two points deserving to be emphasized. First, the findings of this study extended the previous work (Beghetto and Kaufman, 2007; Kaufman and Beghetto, 2009). This study revealed that homework creativity had two typical characteristics, including the personal meaning of students (as represented by the content of items of the HCBS) and the small size of “creativity” and limited in the scope of exercises (small correlations with general creativity). These characteristics are in line with what Mini-C described by the previous studies (Beghetto and Kaufman, 2007; Kaufman and Beghetto, 2009). Second, this study deepened our understanding of the relationship between learning (homework is a part of learning) and creativity which has been discussed more than half a century. One of the main viewpoints is learning and creativity share some fundamental similarities, but no one explained what is the content of these “fundamental similarities” (e.g., Gajda et al., 2017). This study identified one similarity between learning and creativity in the context of homework, that is homework creativity. Homework creativity has the characteristics of homework and creativity at the same time which served as an inner factor in which homework promote creativity.

### The practical implications

The findings in this study also have several potential practical implications. First, homework creativity should be a valuable goal of learning, because homework creativity may make contributions to academic achievement and general creativity simultaneously. They accounted for a total of 10.7% variance of academic achievement and general creativity which are the main goals of learning. Therefore, it is valuable to imbed homework creativity as a goal of learning, especially in the Chinese society (Zheng, 2013).

Second, the items of the HCBS can be used as a vehicle to help students how to develop about homework creativity. Some studies indicated that the creative performance of students will improve just only under the simple requirement of “to be creative please” (Niu and Sternberg, 2003). Similarly, some simple requirements, like “to do your homework in an innovative way,” “don’t stick to what you learned in class,” “to use a simpler method to do your homework,” “to use your imagination when you do homework,” “to design new problems on the basis what learnt,” “to find your own unique insights into your homework,” and “to find multiple solutions to the problem,” which rewritten from the items of the HCBS, can be used in the process of directing homework of students. In fact, these directions are typical behaviors of creative teaching (e.g., Soh, 2000); therefore, they are highly possible to be effective.

Third, the HCBS can be used to measure the degree of homework creativity in ordinary teaching or experimental situations. As demonstrated in the previous sections, the reliability and validity of the HCBS were good enough to play such a role. Based on this tool, the educators can collect the data of homework creativity, and make scientific decisions to improve the performance of people’s teaching or learning.

### Strengths, limitations, and issues for further investigation

The main contribution is that this study accumulated some empirical knowledge about the relationship among homework creativity, homework completion, academic achievement, and general creativity, as well as the psychometric quality of the HCBS. However, the findings of this study should be treated with cautions because of the following limitations. First, our study did not collect the test–retest reliability of the HCBS. This makes it difficult for us to judge the HCBS’s stability over time. Second, the academic achievement data in our study were recorded by self-reported methods, and the objectivity may be more accurate. Third, the lower reliability coefficients existed in two dimensions employed, i.e., the arrange environment of the HMS (the α coefficient was 0.63), and the adventure of the WCAP (the α coefficient was 0.61). Fourth, the samples included here was not representative enough if we plan to generalize the finding to the population of middle and high school students in main land of China.

In addition to those questions listed as laminations, there are a number of issues deserve further examinations. (1) Can these findings from this study be generalized into other samples, especially into those from other cultures? For instances, can the reliability and validity of the HCBS be supported by the data from other samples? Or can the grade effect of the score of the HCBS be observed in other societies? Or can the correlation pattern among homework creativity, homework completion, and academic achievement be reproduced in other samples? (2) What is the role of homework creativity in the development of general creativity? Through longitudinal study, we can systematically observe the effect of homework creativity on individual’s general creativity, including creative skills, knowledge, and motivation. The micro-generating method (Kupers et al., 2018) may be used to reveal how the homework creativity occurs in the learning process. (3) What factors affect homework creativity? Specifically, what effects do the individual factors (e.g., gender) and environmental factors (such as teaching styles of teachers) play in the development of homework creativity? (4) What training programs can be designed to improve homework creativity? What should these programs content? How about their effect on the development of homework creativity? What should the teachers do, if they want to promote creativity in their work situation? All those questions call for further explorations.

## Conclusion

Homework is a complex thing which might have many aspects. Among them, homework creativity was the latest one being named (Guo and Fan, 2018). Based on the testing of its reliability and validity, this study explored the relationships between homework creativity and academic achievement and general creativity, and its variation among different grade levels. The main findings of this study were (1) the eight-item version of the HCBS has good validity and reliability which can be employed in the further studies; (2) homework creativity had positive correlations with academic achievement and general creativity; (3) compared with homework completion, homework creativity made greater contribution to general creativity, but less to academic achievement; and (4) the score of homework creativity of high school students was lower than that of middle school students. Given that this is the first investigation, to our knowledge, that has systematically tapped into homework creativity, there is a critical need to pursue this line of investigation further.

## Data availability statement

The raw data supporting the conclusions of this article will be made available by the authors, without undue reservation.

## Ethics statement

The studies involving human participants were reviewed and approved by the research ethic committee, School of Educational Science, Bohai University. Written informed consent to participate in this study was provided by the participants’ legal guardian/next of kin.

## Author contributions

HF designed the research, collected the data, and interpreted the results. YM and SG analyzed the data and wrote the manuscript. HF, JX, and YM revised the manuscript. YC and HF prepared the HCBS. All authors read and approved the final manuscript.
