# Supplementary material for: Effects of homework creativity on academic achievement and creativity disposition: Evidence from comparisons with homework time and completion based on two independent Chinese samples
Source: Front Psychol. 2022 Aug 12;13:923882. doi: 10.3389/fpsyg.2022.923882 (PMC9417817; doi:10.3389/fpsyg.2022.923882)
Supplement: Supplementary file 1 [file Table_1.DOCX]

Table s1 Distributions of education level of parents and comparisons with the national sample

|  | National sample ^a^ | |  | sample 1 | | | | |  | sample 2 | | | | |
| --- | --- | --- | --- | --- | --- | --- | --- | --- | --- | --- | --- | --- | --- | --- |
|  | total | |  | father | |  | mother | |  | father | |  | mother | |
|  | frequency | Percentage |  | frequency | Percentage |  | frequency | Percentage |  | frequency | Percentage |  | frequency | Percentage |
| no education | 284 | 1.5 |  | 3 | 0.47 |  | 7 | 1.10 |  | 3 | 0.42 |  | 3 | 0.42 |
| elementary | 2880 | 15.5 |  | 42 | 6.57 |  | 48 | 7.51 |  | 64 | 9.01 |  | 55 | 7.75 |
| middle school | 8121 | 43.8 |  | 232 | 36.48 |  | 226 | 35.59 |  | 310 | 43.72 |  | 341 | 48.16 |
| high school | 3994 | 21.6 |  | 216 | 33.96 |  | 199 | 31.34 |  | 238 | 33.57 |  | 208 | 29.38 |
| college | 3089 | 16.67 |  | 140 | 22.01 |  | 155 | 24.41 |  | 84 | 11.85 |  | 95 | 13.42 |
| graduate | 157 | .8 |  | 6 | 0.94 |  | 4 | 0.63 |  | 11 | 1.55 |  | 8 | 1.13 |
| total | 18525 |  |  | 639 |  |  | 639 |  |  | 710 |  |  | 710 |  |
| χ^2^/(5) |  |  |  |  | 8.76 |  |  | 7.46 |  |  | 6.05 |  |  | 5.2 |
| *p* |  |  |  |  | 0.12 |  |  | 0.19 |  |  | 0.30 |  |  |  |

Note. a, data was adopted from China Education Panel Survey (Sun, Shafiq, McClure, & Guo, 2020).

**References**

Sun, L., Shafiq, M. N., McClure, M., & Guo, S. (2020). Are there educational and psychological benefits from private supplementary tutoring in Mainland China? Evidence from the China Education Panel Survey, 2013–15. *International Journal of Educational Development, 72*, 102144. https://doi.org/10.1016 / j.ijedudev.2019.102144

Table s2 Measurement Invariance Unstandardized Parameter Estimates

Step1, configural invariance

|   **Grade 7** |   **Grade 8** |
| --- | --- |
|   **Grade 10** |   **Grade 11** |

Step 2, Factor loading invariance

|   **Grade 7** |   **Grade 8** |
| --- | --- |
|   **Grade 10** |   **Grade 11** |

Step 3, Intercept invariance

|   **Grade 7** |   **Grade 8** |
| --- | --- |
|   **Grade 10** |   **Grade 11** |

Step 4, Residual Variance invariance

|   **Grade 7** |   **Grade 8** |
| --- | --- |
|   **Grade 10** |   **Grade 11** |

Table s3 the full version of HCBS

| Directions: According to your real practice of homework, please choose a suitable number on the right side of the sentences listed.  The meaning of the numbers are: 1= totally disagree, 2 = partially disagree, 3 = uncertain, 4 = partially agree, 5 = totally agree. | | | | | |
| --- | --- | --- | --- | --- | --- |
| items | totally disagree |  |  |  | totally agree |
| 1. I do my homework in an innovative way | 1 | 2 | 3 | 4 | 5 |
| 2. I do my homework without sticking to what I have learned in class | 1 | 2 | 3 | 4 | 5 |
| 3. I found a better solution to complete homework | 1 | 2 | 3 | 4 | 5 |
| 4. I use a simpler method to do the homework | 1 | 2 | 3 | 4 | 5 |
| 5. My rich imagination can be reflected in my homework | 1 | 2 | 3 | 4 | 5 |
| 6. I designed new problems on the basis of teachers | 1 | 2 | 3 | 4 | 5 |
| 7. I designed a neat, clean and clear homework format by myself c | 1 | 2 | 3 | 4 | 5 |
| 8. I have my own unique insights into homework | 1 | 2 | 3 | 4 | 5 |
| 9. I give multiple solutions to a problem | 1 | 2 | 3 | 4 | 5 |

Note. item 7 has been deleted because of its high value of MI, and low link with creativity in its substantive content.
